# Introduction to Language Development in Children: Description to Detect and Prevent Language Difficulties

**DOI:** 10.3390/children9030412

**Published:** 2022-03-14

**Authors:** Eva Aguilar-Mediavilla, Miguel Pérez-Pereira, Elisabet Serrat-Sellabona, Daniel Adrover-Roig

**Affiliations:** 1 Institute of Educational Research and Innovation, Universitat de les Illes Balears, 07122 Palma, Spain; daniel.adrover@uib.es; 2 Department of Developmental and Educational Psychology, Faculty of Psychology, University of Santiago de Compostela, 15705 Santiago de Compostela, Spain; miguel.perez.pereira@usc.es; 3 Department of Psychology, Universitat de Girona, 17004 Girona, Spain; elisabet.serrat@udg.edu

## 1. Introduction

The present Special Issue focuses on studies of language acquisition in children. We particularly addressed the description of language development and the variables affecting it for early detection and prevention of language difficulties. Although language difficulties are very common (14% of children present a primary or secondary language difficulty), these difficulties are misdiagnosed [1,2]. This might be due to the lack of visibility and the scarcity of knowledge in professionals in terms of the long-term consequences of language disorders in education and mental health. To prevent misdiagnosed identification and boost assessment of language difficulties, more typical and atypical language studies are needed. In this sense, a good description of language acquisition could help to detect and prevent language difficulties. Nevertheless, most of the research on child language development has been conducted in English. However, studies in other languages and cross-linguistic studies have shown that some results regarding language development in English may not be transferred into other languages [3]. Despite the increase in the number of studies, there is still a dearth of research on typical and atypical language acquisition in other languages and in bilingual populations. Therefore, this Special Issue aims to fill the current void in these studies, give them visibility, and show the latest research in language acquisition in children.

This Special Issue address child language from different perspectives. In this sense, it includes theoretical and empirical studies on typical and atypical child language acquisition. The contributions include studies about markers of language development in typical development, studies about language development in bilingual populations and several studies about language development in atypical populations including Developmental Language Disorder (DLD), reading disorders, Autism Spectrum Disorder (ASD), preterm children, hearing loss and genetic syndromes.

## 2. Markers of Language Development

Several studies in this Special Issue describe important factors that affect language development at different ages, thus depicting several key aspects to be considered in the prevention of communication and language difficulties throughout childhood. These studies range from the beginnings of word production [4] or gestures [5] to the impact of the use of technological devices in preadolescent children [6]. In addition, they cover different aspects or linguistic components, from phonetics [7], vocabulary [4], or syntax [8], to non-referential gestures related to narrative development [5].

The study by Serrat et al. [4] shows that prelinguistic factors have a greater weight than sociodemographic factors in explaining initial expressive vocabulary. This study shows that children under 18 months of age who imitate more are those who have a greater amount of vocabulary. In a related study, in the Special Issue Rujas et al. [8] shows that imitation or repetition of sentences, as an assessment task, is a useful tool for detecting language difficulties in older children. On the other hand, the study by Liu et al. [7] allows us to observe that the acoustic analysis of the production of certain consonants can provide accurate information on speech development and, therefore, is presented as an aspect to be considered in the evaluation of children’s speech. In their review, Vilà-Giménez et al. [5] note another indicator, not much explored previously, according to which non-referential gestures act as predictors of narrative performance. This suggests that these gestures have important pragmatic functions that help to frame discourse. In older children, the study by Acebedo et al. [6] analyzes a variable that has a negative influence on language development: greater access to and use of media devices. The authors show that preadolescent children who use media devices more frequently and for communication purposes (not for school aid, or to learn new things) present lower language scores, without being influenced by sociodemographic factors.

In short, these studies show the importance of various markers of language development, both as indicators that may be related either to adequate development [4,5,7] or may contribute negatively to language development [6]. On the other hand, repetition of words (imitation) or sentences appears as an indicator of adequate development [4], as well as an important assessment tool to identify difficulties in language development [8].

## 3. Bilingual Development

In terms of bilingual language development, this Special Issue includes two studies. The first study by Kan et al. [9] explores the detection of language impairment in bilingual children by monolingual adults, and the second study by Diaz et al. [10] looks at the mutual longitudinal associations between vocabulary and executive functioning (EF) in monolingual and bilingual children.

As stated, Kan et al. [9] aimed at detecting the risk of language impairment in bilingual children by monolingual adults. The authors focused on how bilingual children’s response speed during a narrative task can serve for categorizing language impairment. To do so, monolingual adults listened to several audio clips from an interactive story-retell task in both Cantonese and in English. Children were six sequential Cantonese-English bilinguals of 4 years of age; three of them had a language impairment and three were TD. Results showed that the interrater reliability was high for both languages, logistic regression and ROC curves revealed that adults were able to identify language impairment in bilingual children by judging their response speed, with higher sensitivity and specificity values in L1 conditions (Cantonese) than in L2 (English). These results highlight the potential relevance of looking at response speed to complement language assessment in bilingual children with language impairment.

Focusing on the potential links between EF and receptive vocabulary, Diaz et al. [10] tested monolingual and bilingual children with 4 years of age on average. The authors used a longitudinal approach with two temporal moments spread one year and departed from the theory of dual language processing as one of the potential sources for the frequently reported gains in EF in bilinguals. Their main goal was to determine whether EF exerted a direct influence on language proficiency or vice versa. Several measures of vocabulary and cognitive flexibility were administered to a sample of bilingual children and to a control group of monolingual preschool children. Results revealed that, only in the monolingual group, vocabulary at moment 1 predicted EF at moment 2. However, EF did not predict vocabulary at moment 2. The authors interpret the lack of longitudinal relations between EF and language abilities in the bilingual group together with the absence of differences in EF between both groups as a potential challenge to the purported advantage in EF in bilinguals and claim for the need of future similar studies.

## 4. Atypical Language Development

The Special Issue included several papers that focused on atypical language development, considering different conditions such as DLD, reading disorders, ASD, preterm, deafness and genetic syndromes.

### 4.1. Developmental Language Disorders (DLD)

DLD, previously named specific language impairment (SLI), is a persistent language delay affecting everyday social interactions or educational progress, in the absence of other biomedical conditions such as ASD, brain injury, hearing loss, genetic conditions or intellectual disability [11,12]. Four of the papers in this Special Issue focused on DLD, evidencing the increasing interest and the need of further studies of this atypical language condition. The works presented covered syntactic processing, lexical and syntactic errors, the use of non-word repetition task as a marker of DLD, and the relation between structural aspects of language, pragmatics, social cognition, and executive functions. 

The work by Roa-Rojas et al. [13] explored a common error in Spanish children with DLD, the gender agreement in clitics, with a real-time processing technique of event-related brain potentials (ERP). Their results evidenced that children with DLD, contrary to their controls, did not show an enhanced anterior negativity between 250 and 500 ms post-target onset when they listened to gender-agreement violations. This result evidences a weaker lexical representation of morphosyntactic gender features in children with DLD.

Additionally, Kornev and Balčiūnienė [14] focused on the grammatical and lexical errors in children with DLD in narrative tasks in Russian. They found that the genre of discourse and age of assessment impacted not only the error distribution in children with DLD, but also in their controls, showing a relation between the cognitive load of the task and the number of errors produced. Their results support the resource deficit model that considers that the DLD is a delay in language performance but not in language competence, with errors being directly influenced by the cognitive demands of utterance and text production.

Following this hypothesis that children with DLD exhibit a limited cognitive load, and thus that language processing can easily overload their cognitive systems, non-word repetition has been proposed as a measure of the phonological working memory capacity and a marker of DLD [15,16]. In this sense, the work of Ahufinger et al. [17] explored the consistency of a non-word repetition task of 3-, 4-, 5- and 6-syllables presented in a random order and with varied wordlikeness ratings. Their results showed that the task discriminated correctly children with and without DLD (from 5 years and 16 years) speaking Catalan–Spanish (bilinguals) and European Portuguese (monolinguals). In this sense, children with DLD were less accurate repeating syllables than typical language developing (TD) children. Interestingly, children with DLD were more accurate repeating non-words with high wordlikeness than low, a pattern that had not been found in TD children. In addition, bilingual children performed worse than monolingual ones. Therefore, this task identified correctly children with DLD and differentiated them from TD children in the three languages (Catalan, Spanish and Portuguese) and in bilingual and monolingual children, making non-word repetition a promising task to detect children with DLD.

The last work in this section, by Andres-Roqueta et al. [18], focused on the association between the results of the parents’ reports in the Children’s Communication Checklist-2 (CCC-2) and several direct-child measures of structural language (phonetics, receptive and expressive grammar, receptive and expressive vocabulary and a composite score), pragmatics (receptive and expressive pragmatics and a composite score), social cognition (strange stories), and executive functions (sustained attention, inhibitory skills and a composite score). The results showed that children with DLD (between 3; 10 and 9 years old) performed worse than their TD peers in all the direct-child measures. The CCC-2 correlated with all direct child assessments in the group of DLD, but only formal measures of structural language predicted parent’s reports in CCC-2. This indicates that CCC-2 answered by parents is a reliable measure to assess formal language, being structural language its best predictor.

### 4.2. Reading and Writing Disorders

Close to DLD and commonly comorbid with this disorder are reading and writing learning disabilities. Reading and writing disabilities are the most prevalent type of learning disabilities, with a prevalence between 7 and 10% and one of the main factors of school failure [19]. It includes impairments in reading decoding (i.e., letter–phoneme correspondence) resulting from problems in phonological processing skills and/or naming problems [20]. Children with RD also show impaired oral language skills, although not as severe as children with DLD [21].

One paper in the present Special Issue focused on reading and writing learning disabilities. González-Valenzuela et al. [22] explored the relationship between the type of delivery (vaginal or caesarean) and the occurrence of learning disabilities in reading (reading accuracy) and writing (phonetic and visual orthography), controlling for several gestational, obstetric, and neonatal variables (maternal age at delivery, gestational age, foetal presentation, Apgar 1, and new-born weight), in six-year-old children born in twin births. Their results showed a relation between the caesarean delivery and the presence of difficulties in reading accuracy, and phonetic and visual orthography. Although the authors advise that more evidence is needed, these findings could be useful in clinical practice to avoid the use of caesarean section on demand or without specialised indication.

### 4.3. Autism Spectrum Disorder (ASD)

Children with ASD show a communication deficit that sometimes is accompanied by formal language difficulties. Two papers in this Special Issue aboard the language and communication deficits in ASD.

One paper in this Special Issue looked at the integration of multimodal information within the communicative setting in toddlers at risk of developing ASD by means of eye-tracking measures. The study by Camero et al. [23] investigated visual attention to establish potential early markers of ASD. A group of 10 age-paired TD children and another group composed of 10 children with an increased likelihood of developing ASD looked at a human face when pronouncing pseudowords on a monitor, which were associated with several pseudo-objects. They found that children with higher odds of developing ASD showed a lower number of fixations to the eyes and larger number of gaze fixations to the mouth than the TD children. ASD children also had a slightly larger non-significant pupil dilation to faces, which was constant during the distinct task periods. They also looked more at the pseudo-object and for a longer time than TD children. In contrast, TD children showed a greater pupil dilation when hearing the pseudowords. The authors discuss that objective measures of eye tracking could be considered as potential markers for early detection of ASD and serve as relevant measures of word processing in both ASD and TD toddlers.

In another paper dedicated to ASD, Torrens and Ruiz [24] explored language and communication in preschool children with ASD compared to other developmental disorders using direct measures and parental reports of language. Results revealed that ASD children show a delay in language comprehension in contrast to language production, together with several problems in non-verbal communication, as compared with children with other developmental disorders. A high association was also observed between participant measures and parental reports of language and communication. These results lead the authors to suggest that complementing participants’ measurements with parental reports is a valuable tool for language assessment. They also suggest that language comprehension deficits and difficulties in non-verbal communication might help diagnostic purposes between children with ASD and children with other neurodevelopmental disorders.

### 4.4. Preterm

Preterm children (very and extremely preterm in particular) are generally considered to present atypical language development. In this Special Issue, two papers are related to this topic. The first one by Pérez-Pereira [25] is a longitudinal study on the prevalence and determinants of language delay carried out with low-risk preterm children. The study spans the period between 10 and 60 months of age, with four measuring points. The participants were grouped into four groups of different gestational ages (GA) corresponding to (1) Extremely and Very preterm, (2) Moderately preterm, (3) Late preterm, and (4) Full-term children. Comparisons of the results obtained in the different language tests indicate that there are hardly any differences between the GA groups in the incidence of language delay (scores below the 10th percentile). The results found suggest that healthy preterm children do not seem to have a higher risk of language delay than full-term children. Logistic regression analyses permitted the identification of those factors that better predicted language delay at different ages, highlighting among these factors, previous language, and cognitive delay.

The second study by Joensuu et al. on the topic of preterm children’s language development [26] investigates the associations between early language development at 2 years and literacy skills at seven years of age, in a sample of 136 very preterm (VP)/very low birth weight children (VLBW) and 137 full-term controls. Their results indicate that lexical production and MLU (Mean Length of Utterances) of the three longest utterances (measured through the Finnish CDI) and the expressive language score (from the Bayley Scales of Infant Development-II) are quite good predictors of prereading skills, reading, and writing at 7 years of age. In addition, most VP/VLBW children who were below the 10th percentile in language measures at 2 years of age had weak literacy skills at 7 years of age.

### 4.5. Deafness

Children with hearing impairment without hearing implants use to show a delay or difficulties in language development. Research with hearing children has revealed that the combination of music (rhyme and rhythm) with phonological awareness activities in intervention programs increments language outcomes. The paper by Holcomb and Wolbers [27] attempts to test the benefits of American Sign Language (ASL) rhyme, rhythm, and phonological awareness for deaf children. An intervention program was provided to five deaf children between 3 and 6 years of age to examine the effects of explicit handshape rhyme awareness instruction on increasing engagement behavior and accuracy in recitation. The findings indicate that recitation skills (although not engagement) in young deaf children can be supported through interventions utilizing ASL rhyme and rhythm supplemented with ASL phonological awareness activities.

### 4.6. Genetic Syndromes

Most genetic syndromes involve cognitive and language developmental impairments. In the present Special Issue, the study by Zanaboni et al. [28] investigates oral motor, speech and language abilities of eight Italian-speaking children (aged 4.6 to 15.4 years) with glucose transporter type 1 deficiency syndrome (GLUT1DS). This syndrome, due to mutations in SLC2A1 gene, implies impaired glucose transport into the brain. Congruently, patients are treated with the ketogenic diet (KD) to meet the energy demands of the developing brain, as it was the case for the participants in this study. The children were assessed with different standardized tests. The results indicated that the patients showed deficits in orofacial praxis, the speech domain, and the language domain (semantic/phonological fluency and receptive grammar, in particular), as well as in the development of several cognitive functions. The authors highlight the importance of a complete speech and language evaluation in GLUT1DS patients to obtain a typical linguistic phenotype, which could guide and improve early diagnosis and intervention.

## 5. Conclusions

The present Special Issue focuses on the major topics of typical and atypical language development with monolingual and bilingual children, covering new and highly innovative studies that have increased the evidence for detecting and preventing language impairment especially in several languages such as Spanish, Catalan, Portuguese, Italian, Russian, Cantonese, Finnish, and American Sign Language.

## Data Availability

Not applicable.
